# Fluoxetine Mimics the Anorectic Action of Estrogen and Its Regulation of Circadian Feeding in Ovariectomized Female Rats

**DOI:** 10.3390/nu12030849

**Published:** 2020-03-22

**Authors:** Yuri Nishimura, Kaori Mabuchi, Natsumi Omura, Ayako Igarashi, Megumi Miura, Nanako Mima, Hiroko Negishi, Keiko Morimoto, Akira Takamata

**Affiliations:** 1 Department of Environmental Health, Nara Women’s University, Kitauoya Nishimachi, Nara 630-8506, Japan; yn92@sussex.ac.uk (Y.N.); mabuchi.kaori@cream.plala.or.jp (K.M.); natsumi.omura@gmail.com (N.O.); ayako.i131@gmail.com (A.I.); mp.blossom.4ever@gmail.com (M.M.); nanako.mima@gmail.com (N.M.); hnegishi16@gmail.com (H.N.); kmorimot@cc.nara-wu.ac.jp (K.M.); 2 Sussex Neuroscience, School of Life Sciences, University of Sussex, Brighton BN1 9QG, UK

**Keywords:** estradiol, selective serotonin reuptake inhibitor, hypophagia, circadian feeding rhythm, suprachiasmatic nucleus

## Abstract

Our previous study demonstrated that chronic estrogen replacement in ovariectomized rats reduces food intake and augments c-Fos expression in the suprachiasmatic nucleus (SCN), specifically during the light phase. Here, we hypothesized that serotonergic neurons in the central nervous system (CNS), which have anorectic action and play a role in regulating circadian rhythm, mediate the light phase-specific anorectic action of estrogen, and that selective serotonin reuptake inhibitors (SSRIs) mimic the hypophagic action of estrogen. Female Wistar rats were ovariectomized and treated with estradiol (E2) or cholesterol by subcutaneously implanting a silicon capsule containing E2 or cholesterol. Then, half of the cholesterol-treated rats were injected with the SSRI fluoxetine (5 mg/kg) (FLX group), while the remaining rats in the cholesterol-treated group (CON group) and all those in the E2 group were injected with saline subcutaneously twice daily at the onsets of the light and dark phases. Both E2 and FLX reduced food intake during the light phase but not the dark phase, and reduced body weight gain. In addition, both E2 and FLX augmented the c-Fos expression in the SCN, specifically during the light phase. These data indicate that FLX exerts estrogen-like antiobesity and hypophagic actions by modifying circadian feeding patterns, and suggest that estrogen regulates circadian feeding rhythm via serotonergic neurons in the CNS.

## 1. Introduction

Estrogen plays an important role in the regulation of energy homeostasis in women [1]. The marked decrease in systemic estrogen levels after menopause often causes weight gain, obesity, or sleep disorder, which are serious public health concerns in modern society [2,3,4,5]. A large number of studies have demonstrated that estrogen reduces energy intake by affecting the activity of hypothalamic neurons that regulate food intake [1,6,7], however the mechanism has not been fully elucidated.

Central serotonin has also been reported to be involved in the regulation of feeding behavior [8]. Selective serotonin reuptake inhibitors (SSRIs), the most commonly prescribed antidepressants, which improve mood by increasing brain serotonin levels, have been shown to suppress food intake in humans and rodents [9,10,11,12]. However, the mode of action of SSRIs regarding feeding behavior has not been fully elucidated.

Findings in previous studies prompted us to hypothesize that estrogen regulates mood and appetite via the central serotonergic system. The hypothesis that the effects of estrogen on feeding behavior are mediated by the serotonergic system is based on the following facts: (1) serotonin, as well as estrogen, has hypophagic and antidepressant actions [13]; (2) serotonergic neurons in the midbrain raphe nucleus express estrogen receptors (ERs) [14]; (3) estrogen increases the expression of the rate-limiting enzyme for serotonin synthesis, tryptophan hydroxylase 2 (tph2) [15], and inhibits serotonin reuptake transporter (SERT) expression [16]; and (4) estrogen increases the expression of some serotonin receptors [17,18]. All of these facts suggest that serotonergic neurons possibly play a role in estrogen-induced hypophagia and mood regulation.

Synchronization of meal times to the environmental day–night cycle is essential for preventing the development of hyperphagia, overweight, or obesity [19,20,21]. In addition, lesions of the suprachiasmatic nucleus (SCN), the master clock in mammals, disrupt the circadian rhythm of food intake and locomotor activity and increase body fat mass [22]. Thus, the regulation of circadian feeding rhythm plays a critical role in energy homeostasis, and its function can be related to neuronal activities in the SCN. Our previous studies have shown that estrogen replacement in ovariectomized rats reduces food intake and augments c-Fos expression in the SCN during the light phase but not the dark phase [23,24]. These findings suggest that circadian regulation of feeding behavior plays a critical role in estrogen-induced hypophagia by altering neuronal activity in the SCN. It has also been reported that central serotonin plays an important role in the regulation of circadian rhythm by modulating the activity of SCN neurons [25], and estrogen enhances the dorsal raphe serotonin system response to environmental light exposure [26]. Furthermore, SSRIs reportedly improve sleep disorder in postmenopausal women, suggesting that the serotonergic system is possibly involved in the circadian rhythm regulation by estrogen [5]. 

In the present study, we hypothesized that estrogen-induced hypophagia is mediated by the central serotonergic system, and therefore that SSRIs mimic the light phase-specific anorectic action of estrogen. To test this hypothesis, we examined the effect of the SSRI fluoxetine (FLX) and estrogen on the circadian pattern of feeding behavior and on the number of c-Fos-like immunoreactive nuclei in the SCN of ovariectomized rats. The data in the present study provide new insights into the anorectic action of FLX and the mechanism underlying estrogen-induced hypophagia.

## 2. Materials and Methods 

### 2.1. Animals and Surgical Procedure

All animal experiments and care procedures were conducted in accordance with the standards relating to the Care, Keeping, and Pain Reduction of Laboratory Animals (Notice of the Ministry of the Environment, Japan). All experimental procedures were approved by the Animal Care and Use Committee of Nara Women’s University (approval numbers 11-04 and 17-01).

Female Wistar rats (Slc:Wistar, Japan SLC, Hamamatsu, Japan) were used in the present experiments. Animals were housed individually in plastic cages with free access to tap water and standard rodent diet (CE2, CLEA Japan, Tokyo, Japan). The room temperature was maintained at 23 °C with 40% relative humidity and a 12 h/12 h light/dark cycle (lights on at 700). The illumination intensity was ~250 lx at the bottom level of the cages throughout the light phase.

All seven-week-old rats were bilaterally ovariectomized from a dorsal approach under general anesthesia (intraperitoneal injection of pentobarbital sodium (50 mg per kg body weight) and 2% sevoflurane), and then a silicon capsule containing 17β-estradiol (E2) or cholesterol was implanted subcutaneously. The capsules were made of silicon tubing (inner length, 20 mm; inner diameter, 2 mm; outer diameter, 3 mm; ARAM Corporation) and were filled with either a mixture of E2 (Sigma-Aldrich Japan, Tokyo, Japan) and cholesterol (Sigma-Aldrich Japan, Tokyo, Japan) powder (1:4 by weight) or cholesterol powder alone. Both ends of the tubing were sealed with silicon-polymer-origin bulking agents (Cemedine Bath Caulk N transparent color, Cemedine Co., Ltd., Tokyo, Japan). After surgery, the rats were given gentamycin to prevent infection.

### 2.2. Experimental Design and Protocol

In the present study, we examined the effects of E2 and FLX on the diurnal feeding pattern and c-Fos expression in the SCN and intergeniculate leaflet (IGL) during the light and dark phases, because the IGL receives environmental photic information from the retina and regulates the activity of SCN via neuropeptide Y (NPY) neurons [27]. 

We also examined the effect of E2 and FLX on depression-like behavior. To examine the effects of E2 and FLX, all rats were ovariectomized and assigned to one of 3 groups: E2-treated rats with saline injection (E2 group), cholesterol-treated rats with FLX injection (FLX group), and cholesterol-treated rats with saline injection (CON group).

Seven days after ovariectomy and capsule implantation, by which time endogenous estrogen effects in cholesterol-treated rats are expected to be eliminated, half of the cholesterol-treated rats were injected subcutaneously with 5 mg/kg FLX (FLX group), while the remaining cholesterol-treated rats (CON group) and all those in the E2 group were injected with the same volume of isotonic saline twice a day for 10 consecutive days. The injections were performed at Zeitgeber times (ZT) of 0 (ZT0) and 12 (ZT12), except for the days with a forced swim test (FST) session.

The diurnal pattern of spontaneous food intake in four rats of each group was measured with an automated feeding monitoring apparatus (FIS-001 and FIC-001; Muromachi, Tokyo, Japan). Data were recorded every minute, and the sum of food consumption every hour and every 12 hours (light phase, 0700–1900; dark phase, 1900–0700) was used for further analysis. Food intake during the light and dark phases in the other rats was measured by manually weighing the food with an electrical balance at ZT0 and ZT12. The average food intake by each rat over 4 days of treatment (3–6 days after the onset of treatment) was calculated and then averaged in each group, because there was no difference in food intake during these four days within the group.

To test whether FLX mimics the antidepressant effect of E2, a FST was performed with Porsolt’s method [28]. The presession occurred on the day just before the onset of FLX treatment, and the test session was performed on the seventh day of FLX or saline treatment. In the presession, rats were individually placed in a transparent plastic cylinder (30 cm diameter, 49 cm high) containing a water column measuring 30 cm in height (24 ± 1 °C) for 15 min. In the test session, the animals were re-exposed to the same conditions as the presession for 5 min. All test sessions were started 60 min after the injection of FLX or saline between 0800 and 1000. Two rats were simultaneously tested, and video images of the swimming behavior of each rat were recorded with two CCD cameras located above and to the side of the swimming pool for subsequent analysis. The immobility time (sec) was analyzed for each animal. In the present study, we defined the immobility time as the time during which more than 80% of the total body surface, as detected by image analysis software (AnyMaze; Stoelting, Wood Dale, IL, USA), was not moving for a consecutive period of 1000 ms.

On day 10 of FLX treatment, the rats were euthanized for immunohistochemical examination of c-Fos expression in the SCN and IGL during the early light (ZT3–ZT7) and dark (ZT15–ZT19) phases. Under deep anesthesia, the rats were perfused transcardially with phosphate buffered saline (PBS) at 4 °C and then with 4% paraformaldehyde in 0.1 M sodium phosphate buffer (PB). Brains were removed and stored in 4% paraformaldehyde solution at 4 °C for postfixation. After 2 days of postfixation, the brains were immersed in 15% sucrose solution diluted with PBS for 24 h and then with 25% sucrose solution for 2 days until they sank. Then, each brain was frozen rapidly at -75 °C (Histo-Tek PINO, Sakura Finetek, Tokyo, Japan) and cut into 30-μm sections with a cryostat microtome at -20 °C (CM 3050S, Leica, Wetzler, Germany). Sections were stored at 4 °C in PBS containing 1% sodium azide.

### 2.3. Immunohistochemistry

Immunohistochemical staining for c-Fos in the SCN and IGL was performed on free-floating sections, as previously reported [23,29]. Briefly, after incubation with rabbit anti-c-Fos antiserum (1:4000; Sc-52, Santa Cruz Biotechnology, Dallas, TX, USA), the sections were incubated with biotinylated goat anti-rabbit IgG (1:400, BA-1000, Vector Laboratories, Burlingame, CA, USA), followed by processing with an avidin-biotinylated horse radish peroxidase complex (1:400; Vectastain ABC kit, Vector Laboratories, Burlingame, CA, USA). Visualization was performed using 0.02% 3,3’-diaminobenzidine (Dojin Laboratories, Kumamoto, Japan) containing 0.01% H_2_O_2_ in 100 mM Tris-HCl buffer (pH 7.4). To visualize the NPY-innervated area in the SCN and NPY neurons in the IGL, we performed double-staining for NPY and c-Fos. 

After staining for c-Fos, the sections were incubated with anti-NPY antibody (1:2000; N9528, Sigma-Aldrich Japan, Tokyo, Japan), followed by incubation with fluorescein isothiocyanate-labeled anti-rabbit IgG (1:400; FI-1000, Vector Laboratories, Burlingame, CA, USA). Finally, the sections were mounted on gelatin-coated slides and cover-slipped with antifade mounting medium (Vectashield®; Vector Laboratories, Burlingame, CA, USA).

To evaluate neural activity levels in the SCN and IGL, c-Fos-immunoreactive (c-Fos-ir) neurons in the SCN and IGL were examined using a microscope (Olympus BX-51; Olympus Corp., Tokyo, Japan). Bright field images of c-Fos and fluorescence images of NPY in the same field were obtained using a cooled CCD camera (Retiga 4000-R, QImaging, Surrey, Canada). The SCN and IGL were identified using the rat brain stereotaxic atlas and sections were carefully matched across all animals in all experimental groups. The number of c-Fos-ir nuclei in the SCN and IGL was counted using ImageJ software (NIH, Bethesda, MD, USA) in two sections in each region of each animal. For the SCN, the number of c-Fos-ir nuclei was counted in the whole SCN and in the ventrolateral region of the SCN. The ventrolateral region was identified as the region in which neuronal terminals were stained with NPY antibody, since the ventrolateral region of the SCN receives NPY neuron projections from the IGL. The regions were easily detected by visualization of NPY fluorescence (Supplementary Figure S1).

### 2.4. Statistics

Data are shown as the mean ± standard error of mean (SEM). One-way analysis of variance (ANOVA) was performed to examine the effects of the treatments on body weight, spontaneous food intake, immobility time during the FST, and c-Fos expression in the SCN and IGL. Differences between groups were determined using Tukey’s post hoc test. Values of *p* < 0.05 were considered statistically significant. 

## 3. Results

### 3.1. Effects of E2 and FLX on Body Weight and Feeding Behavior

Before the FLX treatment experiment, the body weight was 144.4 ± 1.5 g among E2-treated rats and 152.1 ± 1.2 g in cholesterol-treated rats, while body weight gain was 9.2 ± 2.3 g among E2-treated rats and 14.1 ± 0.7 g in cholesterol-treated rats. The body weight and body weight gain before the FLX treatment experiment were significantly smaller among E2-treated rats than cholesterol-treated rats.

**Figure 1:**
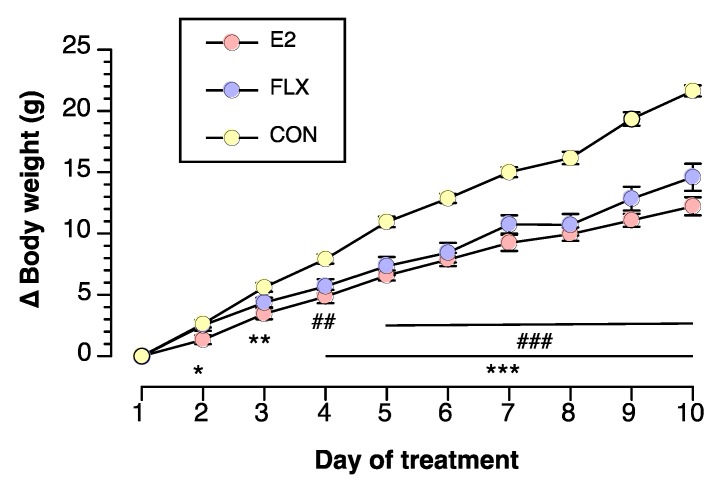
The effect of estradiol replacement or fluoxetine administration on body weight change from the onset of fluoxetine (FLX) treatment in ovariectomized female rats. Female rats were ovariectomized and assigned to the estradiol- or cholesterol-treated group. Six or seven days after ovariectomy, half of the cholesterol-treated rats were subcutaneously (*s.c.*) injected twice daily with 5 mg/kg fluoxetine (FLX) at Zeitgeber times (ZT) of 0 (ZT0) and 12 (ZT12), while the other half of the cholesterol-treated rats (CON) and all of the estradiol-treated rats (E2) were *s.c.* injected with the same volume of saline. Data are shown as the mean ± SEM (*n* = 13 in CON, *n* = 14 in E2, and *n* = 13 in FLX). Note: *, **, and ***: significant differences at *p* < 0.05, *p* < 0.01 and *p* < 0.001, respectively, between the E2 and CON groups. ## and ###: significant differences at *p* < 0.01 and *p* < 0.001, respectively, between the FLX and CON groups.

During the FLX treatment experiment, the body weight gain was less in the FLX and E2 groups than in the CON group, while it was similar in the FLX and E2 groups throughout the experiment (Figure 1).

**Figure 2:**
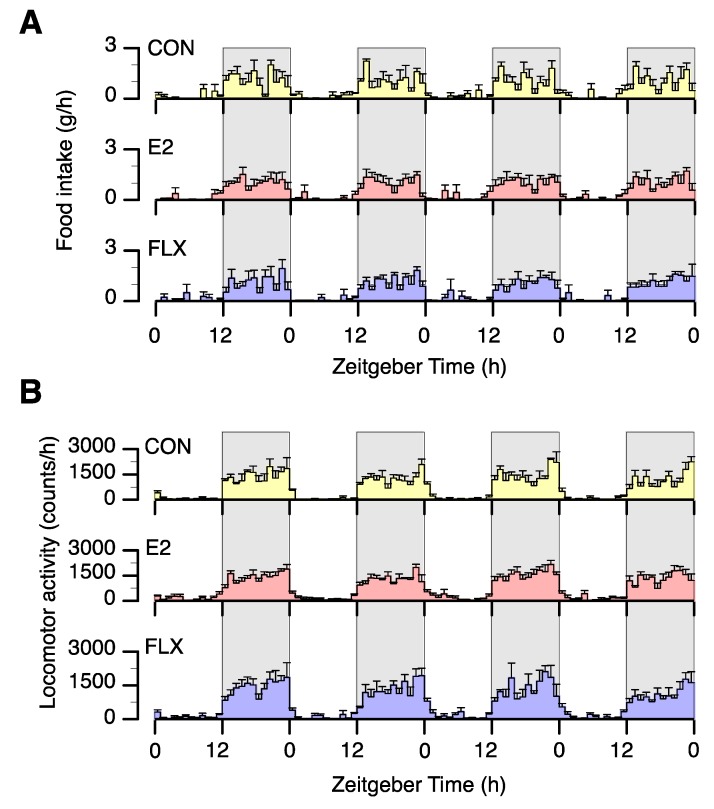
The effect of estradiol (E2) replacement or fluoxetine (FLX) administration on circadian feeding (**A**) and locomotor activity (**B**) patterns for 4 days. Female rats were ovariectomized and assigned to the estradiol- or cholesterol-treated group. Six or seven days after the ovariectomy, a half of the cholesterol-treated rats were subcutaneously (*s.c.*) injected with 5 mg/kg fluoxetine (FLX) twice a day at ZT 0 and 12 every day, while the other half of the cholesterol-treated rats (CON) and all of the estradiol-treated rats (E2) were *s.c.* injected with the same volume of saline.

To examine the effect of FLX or E2 on spontaneous feeding behavior in ovariectomized rats, we first analyzed the circadian feeding pattern and locomotor activity in four of the rats in each group. All animals showed clear circadian rhythms of food intake (Figure 2A) and locomotor activity (Figure 2B), and neither FLX nor E2 induced a phase shift in circadian feeding or locomotor rhythm.

**Figure 3:**
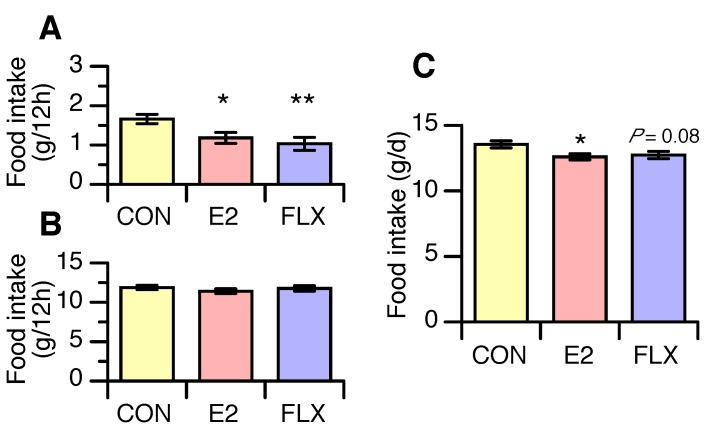
The effect of estradiol (E2) replacement or fluoxetine (FLX) administration on food intake during the light phase (**A**) and dark phase (**B**), and daily food intake (**C**) in ovariectomized female rats. Female rats were ovariectomized and assigned to the estradiol- or cholesterol-treated group. Six or seven days after ovariectomy, half of the cholesterol-treated rats were subcutaneously (*s.c.* injected twice daily with 5 mg/kg fluoxetine (FLX) at ZT0 and ZT12, while the other half of the cholesterol-treated rats (CON) and all of the estradiol-treated rats (E2) were *s.c.* injected with the same volume of saline. Data are shown as the mean ± SEM (*n* = 13 in CON, *n* = 14 in E2, *n* = 13 in FLX). Note: * and **: significant differences at *p* < 0.05 and *p* < 0.01, respectively, compared to the CON group.

Mean food intake over the 4 days during the light phase was lower in the FLX and E2 groups than in the CON group but similar in the FLX and E2 groups (Figure 3A). During the dark phase, however, there were no differences in food intake among treatment groups (Figure 3B). Daily food intake was significantly lower in the E2 group than the CON group and tended to be lower (*p* = 0.08) in the FLX group than in the CON group (Figure 3C).

### 3.2. Effects of E2 and FLX on Depression-Like Behavior

**Figure 4:**
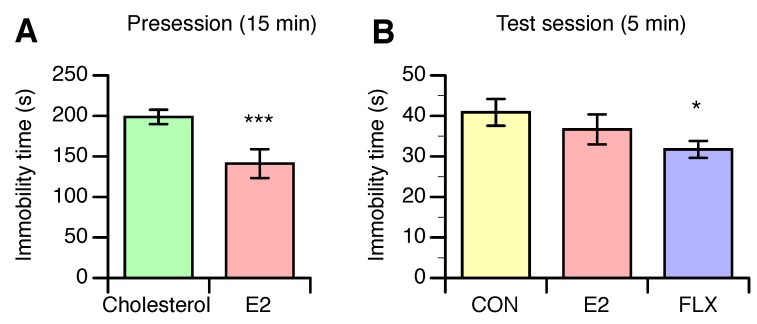
The effect of estradiol replacement in ovariectomized female rats on the immobility time in the presession of Porsolt’s forced swim test (FST) before the fluoxetine treatment (**A**), and effects of estradiol or fluoxetine on the immobility time in the test session of FST (**B**). Female rats were ovariectomized and assigned to the estradiol- or cholesterol-treated group. Six or seven days after ovariectomy, half of the cholesterol-treated rats were subcutaneously (*s.c.*) injected twice daily with 5 mg/kg fluoxetine (FLX) at ZT0 and ZT12, while the other half of the cholesterol-treated rats (CON) and all of the E2-treated rats (E2) were *s.c.* injected with the same volume of saline. The presession was performed before FLX treatment, and the test session was conducted on the seventh day after the onset of daily FLX treatment. Data are shown as the mean ± SEM (*n* = 13 in CON; *n* = 14 in E2; *n* = 13 in FLX). Note: * and ***: significant differences at *p* < 0.05 and *p* < 0.001, respectively, compared to the CON group.

In the presession, which was performed before the FLX treatment experiment, the immobility time was significantly shorter for E2-treated rats than for cholesterol-treated rats (Figure 4A). In the test session, however, the immobility time was not different between the E2 and CON groups, although the time was significantly shorter in the FLX group than in the CON group (Figure 4B).

### 3.3. Effects of E2 and FLX on c-Fos Expression in the SCN and IGL

**Figure 5:**
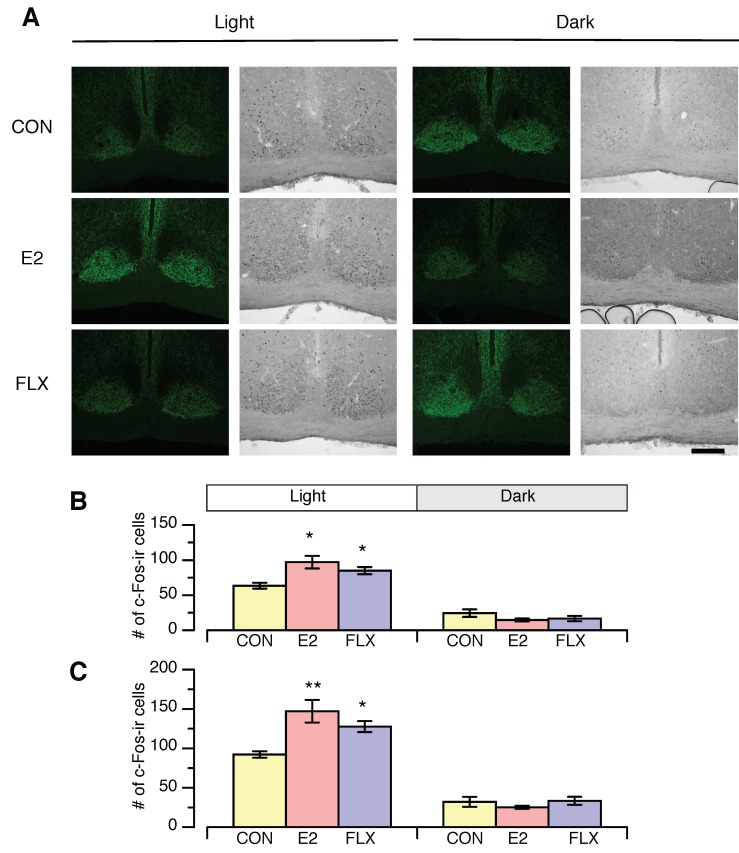
The effect of estradiol or fluoxetine on c-Fos expression in the suprachiasmatic nucleus (SCN) during the light phase (ZT3-7) and dark phase (ZT15-19) in ovariectomized female rats. (**A**): Representative microscope images of c-Fos-immunoreactive (ir) nuclei and neuropeptide Y (NPY) immunoreactivity in the SCN during the light phase and dark phase. Scale bar = 200 µm. (**B**): Number of c-Fos-ir nuclei in the ventrolateral SCN during the light phase and dark phase. (**C**): Number of c-Fos-ir nuclei in the whole SCN during the light phase and dark phase. Female rats were ovariectomized and assigned to the estradiol- or cholesterol-treated group. Six or seven days after ovariectomy, half of the cholesterol-treated rats were subcutaneously (*s.c.*) injected twice daily with 5 mg/kg fluoxetine (FLX) at ZT0 and ZT12, while the other half of the cholesterol-treated rats (CON) and all of the E2-treated rats (E2) were *s.c.* injected with the same volume of saline. Samples were obtained on day 10 of FLX treatment. Data are shown as the mean ± SEM (*n* = 9 in CON, *n* = 8 in E2, and *n* = 8 in FLX during the light phase; *n* = 5 in CON, *n* = 5 in E2, and *n* = 6 in FLX during the dark phase). Note: * and **: significant differences at *p* < 0.05 and *p* < 0.01, respectively, between the CON and treatment groups.

To determine the region of interest, we performed double-staining for c-Fos and NPY (Figure 5A), since the ventrolateral region of the SCN receives NPY neuron projections from the IGL. The regions were easily detected by fluorescence visualization of NPY.

During the light phase, the numbers of c-Fos-ir cells in both the ventrolateral SCN (VL-SCN) and the whole SCN were significantly greater in the E2 and FLX groups than in the CON group (Figure 5A). The numbers of c-Fos-ir cells in both the VL-SCN and the whole SCN were not altered by any treatment during the dark phase (Figure 5B).

**Figure 6:**
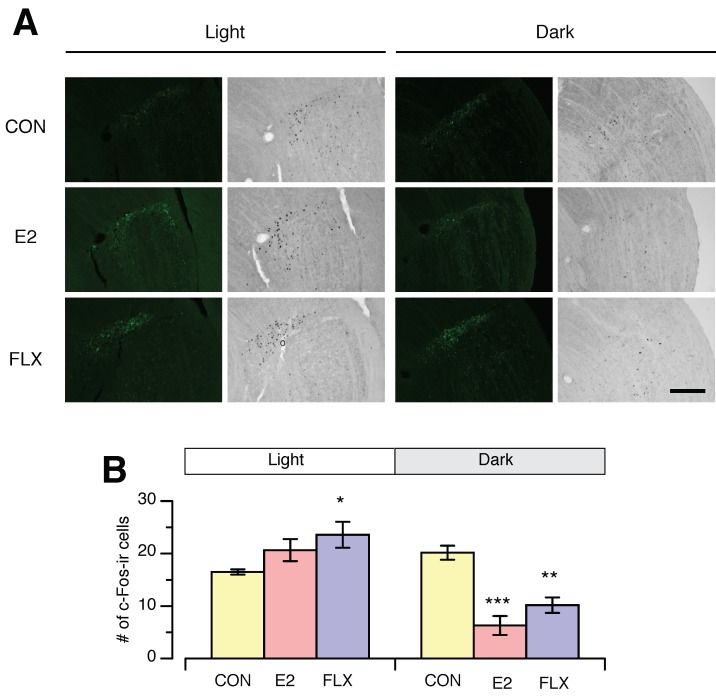
The effect of estradiol or fluoxetine on c-Fos expression in the intergeniculate leaflet (IGL) during the light phase (ZT3-7) and dark phase (ZT15-19) in ovariectomized female rats. (**A**): Representative microscope images of c-Fos-immunoreactive (ir) nuclei and neuropeptide Y (NPY) immunoreactivity in the IGL during the light phase and dark phase. Scale bar = 200 µm. (**B**): Number of c-Fos-ir nuclei in the IGL during the light phase and dark phase. Female rats were ovariectomized and assigned to the estradiol- or cholesterol-treated group. Six or seven days after ovariectomy, half of the cholesterol-treated rats were subcutaneouly (*s.c.*) injected twice daily with 5 mg/kg fluoxetine (FLX) at ZT0 and ZT12, while the other half of the cholesterol-treated rats (CON) and all of the E2-treated rats (E2) were *s.c.* injected with the same volume of saline. Samples were obtained on the 10th of FLX treatment. Data are shown as the mean ± SEM (*n* = 9 in CON, *n* = 8 in E2, and *n* = 8 in FLX during the light phase; *n* = 5 in CON, *n* = 5 in E2, and *n* = 6 in FLX during the dark phase). Note: *, **, and ***: significant differences at *p* < 0.05, *p* < 0.01 and *p* < 0.001, respectively, between the CON and treatment groups.

In the IGL, the number of c-Fos-ir nuclei was higher in the FLX group than in the CON group but similar in the E2 and CON groups during the light phase (Figure 6). In contrast, during the dark phase, the number of c-Fos-ir cells in the IGL was significantly lower in the E2 and FLX groups than in the CON group (Figure 6). Collectively, FLX exerts an estrogen-like action on c-Fos expression in the SCN, which was specific to the light phase, but the effects of FLX and E2 on c-Fos expression in the IGL were not light-phase-specific.

## 4. Discussion

The primary finding of the present study is that FLX exerts an estrogen-like anorectic action. We found in the present study that both E2 and FLX attenuated food intake during the light phase but not the dark phase. Furthermore, similar to E2, FLX enhanced c-Fos expression in the SCN, specifically during the light phase, suggesting that E2- or FLX-induced hypophagia could be associated with enhanced activity of SCN neurons. It is likely that estrogen induces hypophagia during the light phase by modifying the circadian rhythm regulatory system via serotonergic neurons.

We found in the present study that both E2- and FLX-induced hypophagia occurred, specifically during the light phase, and neither E2 nor FLX shifted the phase of feeding rhythm. Thus, both E2 and FLX modify the amplitude of the circadian feeding rhythm without altering the phase of the feeding rhythm. The actions of E2 and FLX, which occurred specifically during the light phase, were not due to the timing of administration, because in the present study E2 was continuously administered throughout the experiment and FLX was injected twice daily at the beginning of the light and dark phases. These results indicate the potential that both E2 and FLX modify the circadian feeding rhythm by altering the activity of the SCN, specifically during the light phase. Similar to our findings in previous studies, we found in the present study that E2 enhanced c-Fos expression in the SCN during the light phase [23,24]. Additionally, the present study revealed that FLX enhanced c-Fos expression in the SCN during the light phase in a similar fashion as E2. It is possible that estrogen enhances the response of the SCN to environmental light via the dorsal raphe serotonin system [26,30].

Electrical lesion of the SCN disrupts the circadian feeding rhythm and induces obesity, suggesting that neurons in the SCN play a critical role in regulating circadian feeding behavior [22]. Thus, our results suggest that E2 or FLX modifies the activity of neurons in the SCN, which possibly modifies circadian feeding rhythm and induces hypophagia.

In the present study, FLX mimicked the E2-induced modifications of the diurnal pattern of feeding behavior and neuronal activity in the SCN. Our findings support the results from previous studies [31,32], showing that an increase in serotonin concentration is possibly involved in estrogen-induced hypophagia. Eckel et al. reported that d-fenfluramine (d-fen), a stimulant of serotonin release from presynaptic vesicles, also attenuated food intake in ovariectomized female rats [11]. Because estrogen enhances serotonin neuron function in the brain [33,34], serotonin may play a role in estrogen-induced hypophagia. Moreover, it is generally accepted that serotonergic neurons in the raphe nucleus express ERs [14]. Additionally, serotonin synthesis in the raphe is affected by the systemic estrogen concentration [15]. These findings strongly suggest that the hypophagic effect of estrogen is mediated by increased serotonin in the synaptic cleft. However, our data were not able to reveal a direct link between the effects of estrogen and serotonin on feeding behavior. Further study is required to elucidate the involvement of serotonin in estrogen-induced hypophagia.

Our data suggest that the serotonergic system is involved in the estrogen-induced hypophagia that occurs specifically during the light phase, but the possibility that FLX directly or indirectly stimulates ERs and exerts estrogen-like effects cannot be ruled out. An in vitro study demonstrated that a low dose of FLX may interact with the ERs [35]. However, the activity of FLX on ERs in the CNS and the site of action of FLX in the CNS remains unknown. Further study is required to elucidate the direct involvement of serotonin in estrogen-induced hypophagia.

We confirmed the hypophagic effect of E2 in the present study. However, the effect was relatively small in the present study compared to the effect in our previous studies [23,24]. It is unclear why the hypophagic effect of E2 was smaller in the present study. One possible reason is that the strain of rats used in the present study (Slc:Wistar) was different from that in the previous studies (Jcl:Wistar). In fact, the daily food intake and food intake during the light phase in estradiol deficit rats (CON) in the present study was smaller than in the previous studies.

Serotonin neurons in the raphe nucleus project into the SCN and regulate neuronal activity in the SCN [27,36,37]. It is likely that both E2 and FLX enhance the activity of serotonergic neurons, which in turn increases neuronal activity in the SCN. Our findings strongly suggest that the estrogen-induced increase in the activity of serotoninergic neurons innervating the SCN augments neuronal activity in the SCN during the light phase. However, it remains unknown whether the alteration in the diurnal feeding pattern induced by E2 and FLX is directly mediated by the SCN. It is also possible that E2 and FLX act on targets outside the SCN that modify SCN neuronal activity and feeding in parallel.

Serotonin neuron terminals are spread widely throughout the central nervous system (CNS) and play roles in various physiological and behavioral functions (e.g., feeding behavior, sleep–wake pattern, and stress-induced responses). Serotonin neurons inhibit orexin neurons in the perifornical hypothalamic area, and orexin neurons play critical roles in the regulation of feeding and diurnal rhythm of sleep–wakefulness [29,38]. We reported that estradiol replacement in ovariectomized rats increased the duration of non-REM sleep during the light phase [39]. These suggest that diurnal feeding and sleep–wakefulness rhythm regulation mediated by orexin neurons via the serotonergic system is possibly involved in the reduced food intake induced by E2 and FLX during the light phase.

Furthermore, both estrogen and serotonin improve depression-like behavior, as evaluated by Porsolt’s FST, and attenuate feeding behavior [13]. In the present study, the characteristics of the antidepressant effect induced by chronic FLX treatment were not similar to those of E2; the depression-like behavior in ovariectomized rats was reduced by FLX but not E2 in the test session, while E2 reduced depression-like behavior in the presession. The inconsistent results between sessions might be derived from the duration of swimming; that is, E2 exerted an antidepressant effect in the 15 min presession but not in the 5 min test session. Another possible reason for the different effects of E2 and FLX on depression-like behavior is the method of evaluating the immobility time; we evaluated the immobility time objectively using software, however the immobility time in the present study was shorter than previously reported. Nevertheless, our results showed that both E2 and FLX likely exert antidepressant effects, although E2 did not show such an effect in the test session. These results suggest that the serotonergic system in the CNS is involved in the estrogen-induced modulation of various physiological and behavioral functions.

In the present study, FLX but not E2 affected c-Fos expression in the IGL during the light phase, while both treatments reduced c-Fos expression in the IGL during the dark phase. It is well established that serotonergic neurons in the raphe nucleus project to IGL neurons, which modulate neuronal activity in the IGL [11]. Additionally, the IGL receives photic input from the retina and sends projections into the SCN, which are important for modulating circadian rhythm in the SCN [40,41]. In addition, neurons in the IGL express ERs [42]. Thus, the IGL is another possible region that mediates estrogen-induced modulation of circadian feeding. In the present study, the diurnal variation in c-Fos expression in the IGL was significantly smaller in the CON group than in the E2 or FLX groups, suggesting that estrogen deficiency likely disrupts the circadian rhythm of neuronal activity in the IGL, but that this effect can be reversed by E2 replacement and FLX. However, the effect of E2 or FLX on neural activity in the IGL did not occur specifically during the light phase, suggesting that the IGL is unlikely to be directly involved in E2- or FLX-induced hypophagia, which occurs specifically during the light phase.

Our data suggest that the serotonergic system is involved in the estrogen-induced increase in c-Fos expression in the SCN that occurs specifically during the light phase, but it is also possible that other mechanisms independent of the serotonergic system are involved in the estrogen-induced increase in neural activity of the SCN. Pacemaker cells of the SCN preferentially express ERβ, and a recent study revealed that the ER-β promoter has an enhancer box (E-box) sequence [43], which is a DNA response element that regulates gene expression and is essential for establishing the circadian feedback loop. The expression of ER-β might fluctuate over a day, and this expression pattern could result in the light-phase-specific effects of estrogen. Thus, it is also possible that different mechanisms are involved in the E2- and FLX-induced increases in c-Fos expression in the SCN.

## 5. Conclusions

The present data revealed that FLX mimics the anorectic and antiobesity actions of estrogen and its ability to enhance neuronal activity in the SCN, which occurs specifically during the light phase. The data suggest that estrogen-induced anorexia is possibly mediated by the serotonergic system. The data also suggest that the altered neuronal activity in the SCN induced by estrogen via the serotonergic system is a potential mechanism for the estrogen-induced hypophagia that occurs specifically during the light phase. However, the neural network involved in linking the activity of the SCN and neurons regulating feeding behavior remains to be elucidated.

## Supplementary Materials

The following are available online at https://www.mdpi.com/2072-6643/12/3/849/s1. Figure S1: Identification of ventrolateral and dorsolateral regions of the sprachiasmatic nucleus, and intergenuculate leaflet with neuropeptide Y immunofluorescence image.
