# Supplementary material for: Fluoxetine Mimics the Anorectic Action of Estrogen and Its Regulation of Circadian Feeding in Ovariectomized Female Rats
Source: Nutrients. 2020 Mar 22;12(3):849. doi: 10.3390/nu12030849 (PMC7146435; doi:10.3390/nu12030849)
Supplement: Supplementary file 1 [file nutrients-12-00849-s001.pdf]

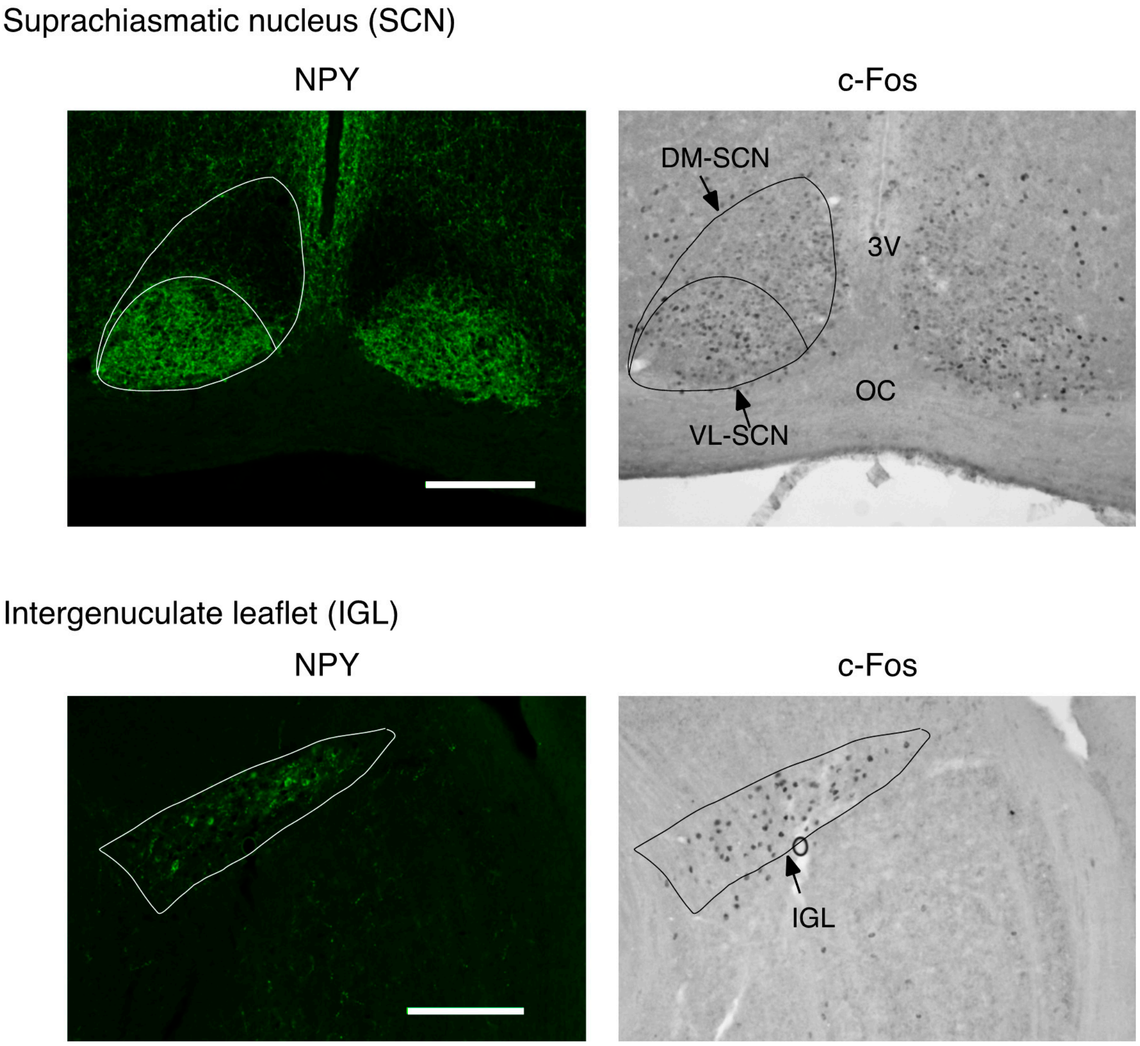

Figure S1. Identification of ventrolateral and dorsolateral regions of the sprachiasmatic nucleus, and intergenuculate leaflet with neuropeptide Y immunofluorescence image. Microscopic images of neuropeptide Y (NPY) fluorescent-labelled immunoreactive region and c-Fos immunoreactive nuclei in the suprachiasmatic nucleus (SCN; top) and intergenuculate nucleus (IGL; bottom) of the same section. DM-SCN, dorsomedial SCN; VL-SCN, ventrolateral SCN; 3V, third ventricle; OC, optic chiasma. Scale bar = 200 μm. We determined the area of the VL-SCN as the area in which neuronal terminals stained with NPY antibody are densely distributed, and the area of the intergenuculate leaflet as the area in which neurons stained with NPY antibody are densely located.
